# Structural insights into the mechanism of RNA recognition by the N-terminal RNA-binding domain of the SARS-CoV-2 nucleocapsid phosphoprotein

**DOI:** 10.1016/j.csbj.2020.08.006

**Published:** 2020-08-12

**Authors:** Abbas Khan, Muhammad Tahir Khan, Shoaib Saleem, Muhammad Junaid, Arif Ali, Syed Shujait Ali, Mazhar Khan, Dong-Qing Wei

**Affiliations:** a State Key Lab of Microbial Metabolism, Department of Bioinformatics and Biological Statistics, School of Life Sciences and Biotechnology, Shanghai Jiao Tong University, Shanghai 200240, China; b National Center for Bioinformatics, Quaid-i-Azam University, 45320 Islamabad, Pakistan; c Center for Biotechnology and Microbiology, University of Swat, Swat, Khyber Pakhtunkhwa, Pakistan; d The CAS Key Laboratory of Innate Immunity and Chronic Diseases, Hefei National Laboratory for Physical Sciences at Microscale, School of Life Sciences, CAS Center for Excellence in Molecular Cell Science, University of Science and Technology of China (USTC), Collaborative Innovation Center of Genetics and Development, Hefei 230027, Anhui, China; e State Key Laboratory of Microbial Metabolism, Shanghai-Islamabad-Belgrade Joint Innovation Center on Antibacterial Resistances, Joint Laboratory of International Cooperation in Metabolic and Developmental Sciences, Ministry of Education and School of Life Sciences and Biotechnology, Shanghai Jiao Tong University, Shanghai 200030, China; f Peng Cheng Laboratory, Vanke Cloud City Phase I Building 8, Xili Street, Nashan District, Shenzhen, Guangdong 518055, China; g Department of Bioinformatics and Biosciences, Capital University of Science and Technology, Islamabad, Pakistan

**Keywords:** Nucleocapsid Phosphoprotein, RNA binding, Alanine scanning, PCA, DCCM, Free energy calculations

## Abstract

The emergence of recent SARS-CoV-2 has become a global health issue. This single-stranded positive-sense RNA virus is continuously spreading with increasing morbidities and mortalities. The proteome of this virus contains four structural and sixteen nonstructural proteins that ensure the replication of the virus in the host cell. However, the role of phosphoprotein (N) in RNA recognition, replicating, transcribing the viral genome, and modulating the host immune response is indispensable. Recently, the NMR structure of the N-terminal domain of the Nucleocapsid Phosphoprotein has been reported, but its precise structural mechanism of how the ssRNA interacts with it is not reported yet. Therefore, here, we have used an integrated computational pipeline to identify the key residues, which play an essential role in RNA recognition. We generated multiple variants by using an alanine scanning strategy and performed an extensive simulation for each system to signify the role of each interfacial residue. Our analyses suggest that residues T57A, H59A, S105A, R107A, F171A, and Y172A significantly affected the dynamics and binding of RNA. Furthermore, per-residue energy decomposition analysis suggests that residues T57, H59, S105 and R107 are the key hotspots for drug discovery. Thus, these residues may be useful as potential pharmacophores in drug designing.

## Graphical abstract

**Figure:**
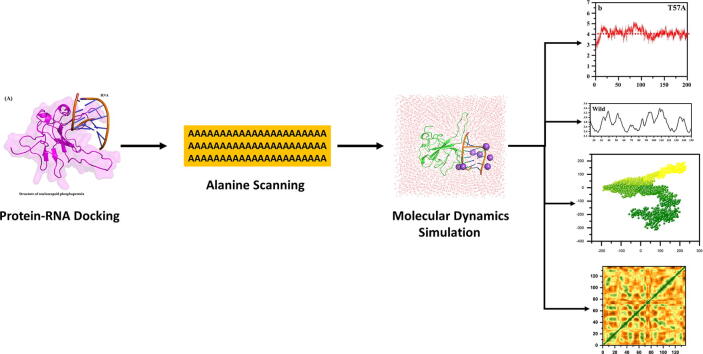

## 1 Introduction

SARS-CoV-2 belongs to the single-stranded positive-sense RNA family. This virus family has a large genome (30 kb RNA genome) that encodes four structural proteins, small envelope (E), matrix (M), nucleocapsid phosphoprotein (N), spike (S), and sixteen nonstructural proteins (nsp1-16) that together, ensure replication of the virus in the host cell [1]. The non-structural proteins, mostly associated with RNA replication, carry out the enzymatic function required for viral replication. The genome of SARS-CoV-2 also encodes for nsp7, nsp8, and nsp12 that together form a complex called RNA-dependent RNA-polymerase, nsp10, nsp13, nsp14, and 16 complexes called RNA capping machinery, and nsp3, 3PLpro, and nsp5 known as proteases that impede innate immunity and also essential for cleaving viral polyproteins [2], [3].

The first 66.66% part (two-thirds) of SARS-CoV-2 genome is known as ORF1a/b region and encodes for the non-structural proteins, whereas the remaining one-third part of genome encodes the accessory proteins and four structural proteins [4]. In recent antiviral drug and vaccine designing investigations spike proteins (S) and proteases were targeted. However, the mutations in spike protein would be helpful to evade the effect of these drugs. On other hand the use of protease inhibitors can harm the homologous cellular proteases [5], [6]. Therefore, it is essential to investigate novel targets and devise comprehensive strategies for the protection of human against all sort of viral encroachment including acute respiratory infection caused SAR-CoV-2.

In corona viruses the multifunctional N protein is essential for transcription as well as replication. N protein binds to the viral genome and contributes in packing it to get long helical nucleocapsid structure [7], [8], [9]. Previous studies indicated the involvement of N protein in host-pathogen interactions by regulating apoptosis, actin reorganization and host cell cycle progression [10], [11]. The highly immunogenic nature and most expressed protein during infection make N protein a valuable novel target for devising novel strategies to combat respiratory infections caused by CoV. The recent studies suggested that the N proteins (homologous in different coronaviruses) is composed of five different domains and parts: the N terminal flexible arm, the N terminal domain (NTD), the central disordered region (LKR, (Ser/Arg (SR)-rich linker), the C terminal domain (CTD) and the C terminal flexible tail [3]. The three intrinsically disordered proteins or regions (IDPs or IDRs), the N terminal flexible arm), the central disordered region (LKR, (Ser/Arg (SR)-rich linker) and the C terminal flexible tail are flexible [3]. These IDRs plays vital role in macromolecules interactions [3]. Diverse studies highlighted the involvement of NTD in RNA binding, (SR)-rich linker in primary phosphorylation and CTD in oligomerization respectively [11].In N terminal of coronavirus N protein several residues associated with RNA binding and infectivity has been identified [12], [13], [14]. However, N protein of SAR-CoV-2 required further investigation to confirm the previous findings in other corona viruses. The N-terminal RNA binding domain (N-NTD) captures the RNA genome [15], [16], [17]. In contrast, the C-terminal domain anchors the ribonucleoprotein complex to the viral membrane via its interaction with the M protein [18]. The four structural proteins, together with the viral + RNA genome and the envelope, constitute the complete virion [16], [17], [19]. Both of these domains have the RNA binding affinity, while the CTD binds the M protein, establishing the physical linkage between the envelope and +RNA. The SARS N proteins also play regulatory roles in the viral life cycle through the host intracellular machinery. A more recent study shows the structure of N protein, right hand-like fold, composed of a β-sheet core with an extended central loop. The core region adopts a five-stranded U-shaped right-handed antiparallelβ-sheet platform with the topology β4-β2-β3-β1-β5, flanked by two short α-helices. A prominent feature of the structure is a large extending loop between β2-β3 that forms a long basic β-hairpin (β2′ and β3′) [15].

Since the role of Nucleocapsid Phosphoprotein to recognize the RNA is crucial [9]. It binds the viral RNA genome and packs them into a complex of ribonucleoprotein (RNP). This RNP complex is critical for retaining highly ordered RNA conformation apt for replicating and transcribing the viral genome [3]. This complex is also being required for host-pathogen interactions regulation, a highly immunogenic and abundantly expressed protein during infection [8].

The NMR structure of the SARS-CoV-2N-terminal and C-terminal domains of nucleocapsid phosphoprotein has recently been reported but the role of N-terminal domain in recognizing the RNA is not clear [15]. The N-terminal domain reported is a monomer structure and does not contain the interacting RNA. Since it is important to understand the interaction mechanism to provide a way in the treatment of recent pneumonia. Herein, we combined multiple computational approaches to understand how the RNA interacts with this nucleocapsid phosphoprotein. We used computational docking approaches to understand the role of critical residues in interaction with RNA. Furthermore, we used the in-silico mutagenesis strategy to determine the impact of each residue taking part in the interaction. We also performed molecular dynamics simulation, binding free energy calculations, Dynamics cross-correlation analysis, principal component analysis, and Free energy landscape to deeply understand the role RNA recognition mechanism by the nucleocapsid phosphoprotein. The findings of this research can be useful and will provide a better understanding of rapid drug designing to control the global epidemic of SARS-CoV-2.

## 2 Methods

### 2.1 Nucleocapsid phosphoprotein retrieval and preparation

For docking studies, the recently submitted the solution NMR structure of the SARs-CoV-2 nucleocapsid phosphoprotein (PDB ID: 6YI3**)** was extracted from Protein Data Bank [20]. The structure was subjected to preparation by Protein Preparation Wizard in Molecular Operating Environment (MOE) [21]. The missing hydrogens were added, and partial charges were assigned. The structure was also analyzed for structural breaks and unknown residues.

### 2.2 Docking of nucleocapsid phosphoprotein and RNA

Prior to docking, the 3D structure of RNA was constructed by using the sequences reported by a recent study [15]. The structure was generated and analyzed for topology defects. All the grooves were carefully examined before the docking. The NMR structure of the N-terminal nucleocapsid phosphoprotein was retrieved from RCSB databank. For the docking, we used multiple algorithms. HADDOCK (High Ambiguity Driven protein–protein Docking) [22] that makes use of biochemical and biophysical interaction data such as chemical shift perturbation data resulting from NMR titration experiments or mutagenesis data and Ambiguous Interaction Restraints (AIRs) to drive the docking process. We used the Guru interface to predict the docking poses, which is considered as the best interface among all the four interfaces owned by the HADDOCK server. Guru interface has all the available (approximately 500) features for protein-RNA/DNA docking. The best structural complex was obtained based on the default parameter (lowest intermolecular energies). To get the best results, we also performed the docking of RNA with Nucleocapsid Phosphoprotein using NPDock [23], which is an online server for protein-nucleic acid docking. NPDock uses scoring of poses, clustering of the best-scored models, and refinement of the most promising solutions to give the best results. The best scoring complex was retrieved from NPDock and analyzed. A comparative analysis of the best complexes was performed to process the best compounds for further analyses. For interaction analysis, DNAproDB [24] was used, which provides an automated structure-processing pipeline to extract structural features from DNA-nucleic acid complexes.

### 2.3 Alanine scanning (mutagenesis)

Alanine scanning is a site-directed mutagenesis method used to identify whether a particular residue contributes to the stability or function of a specific protein. Alanine is used owing to its chemically inert, non-bulky, methyl functional group that nevertheless imitates the secondary structure preferences that certain other amino acids exhibit. This strategy also can be used to discern if the side chain of a particular residue plays an important role in bioactivity or not [25], [26]. Mutagenesis [27] was performed using MOE (Molecular Operating Environment) [21]that computes the particular amino acid residue impact upon replacing by alanine. The complete procedure of alanine scanning mutagenesis has been given in the previous study [28]. Two parameters dAffinity and dStability were considered while calculating the impact of alanine substitutions. High positive dAffinity and dStability means highly significant substitution. Furthermore, we also used mCSM-NA an online server, to determine the impact of alanine substitution on the structure and affinity of nucleocapsid phosphoprotein-RNA complex. mCSM–NA [29] uses the graph-based signature concept, which combines a pharmacophore modeling and information of nucleic acid properties to predict and characterize the effect of a single point missense mutation on protein-nucleic acid binding. To further validate our results, we also used DrugScorePPI [30] an online webserver based on the knowledge-based scoring function to predict changes in the binding free energy upon alanine mutations. Combining these three methods predicted the most significant substitutions for RNA interaction with the binding protein.

### 2.4 Molecular dynamics (MD) simulation

The WT and mutant type complex were subjected to molecular dynamics (MD) simulation studies using the Amber package [31]. The TIP3P water model was used, and the system was neutralized by Na^+^ counter ions addition. The OL3 force field was used for RNA. The system was energy minimized by using the steepest descent algorithm. Restraining simulation of the position was employed to equilibrate the system and solvent around the protein before the actual simulation. In a constant number of atoms, volume, pressure, and temperature (NPT and NVT), ensembles were applied to the system for the MD simulation studies. Particle Mesh Ewald (PME) SHAKE algorithm was used for hydrogen interactions [32]. A total of 400 ns of MD simulation for each system was performed and repeated three times. CPPTRAJ and PYTRAJ [33] was used for RMSD, RMSF, and other analysis of the MD trajectories. Pymol was used for visualization [34]. Furthermore, we also calculated the total energies of all the systems including wild type and mutants.

### 2.5 Unsupervised clustering of MD trajectories and free energy landscape

Principal Component Analysis (PCA) [35], [36] was used to obtain the internal motion of the system. A CPPTRAJ package in Amber was used for this function. The positional covariance matrix for eigenvectors and its atomic coordinates were calculated. The diagonal matrix of eigenvalues was obtained by diagonalizing the matrix with the help of orthogonal coordinate transformation. The principal components were obtained based on eigenvalues and eigenvectors, which highlighted the motion of trajectories during simulation [37], [38]. The first two principal components, known as PC1 and PC2, were used to calculate the free energy landscape (FEL) in the following equation.

$$\Delta G\left(X\right)=-K_{B}TlnP\left(X\right)$$

where X indicates the response of the two principal components, KB is Boltzmann constant, and P(X) is the dispersion of the framework’s likelihood on the first two principal components.

### 2.6 Dynamic cross-correlation

A time subordinate movements of Cα atoms was obtained by using dynamics cross-correlation maps (DCCM) approach [39]. Thus to understand the correlated and anti-correlated motions of C-α atoms of all the systems residues, correlation matrix was obtained. The following equation was used for DCCM calculations.

$$C_{\mathrm{ij}}=\langle \Delta r_{i}\times \Delta r_{j}\rangle /{\left(\langle \Delta r_{i}^{2}\rangle \langle \Delta r_{j}^{2}\rangle \right)}^{2}$$

The matrix (*Cij*) represents the time-correlated data of protein between the *i* and *j* atoms. Cα atoms from the 20,000 snapshots were chosen to construct the matrix at 0.002 ns intervals. In the plot, the positive values specify correlated motions, whereas negative values indicate anti-correlated motion during the simulation.

### 2.7 Binding free energy calculations

The MMGBSA method was used to calculate the free energy of binding between WT and MTs complexes [40]. A total of 20,000 conformations extracted from the 400 ns trajectories of 0.2 ns time intervals were used in the calculation. Mechanics Poisson–Boltzmann surface area (MM/PBSA) and Molecular Mechanics/Generalized Born Surface Area (MM/GBSA) are two efficient approaches to analyze the free energy. The values of MM/PBSA are significantly in correlation with experimental approaches [41]. MM/PBSA has been extensively applied in protein–protein interaction and protein–ligand binding. Here, we used both MMPBSA and MMGBSA approaches to calculate the binding free energy.

For Free Energy calculation the following equation was used:

ΔG(bind)=ΔG(complex)-[ΔG(receptor)+ΔG(ligand)]

Each component of the total free energy was estimated using the following equation:

$$G=G_{bond}+G_{ele}+G_{vdW}+G_{pol}+G_{npol}-TS$$

where G_bond_, G_ele_, and G_vdW_ denotes bonded, electrostatic, and van der Waals interactions, respectively. G-pol and G_npol_ are polar and nonpolar solvated free energies. The G_pol_ and G_npol_ are calculated by the generalized Born (GB) implicit solvent method with the solvent-accessible surface area SASA term. Furthermore, we also performed per-residues energy decomposition analysis to understand the energy contribution of each residue to the whole energy.

## 3 Results

### 3.1 Interaction of nucleocapsid phosphoprotein with RNA

**Fig. 1:**
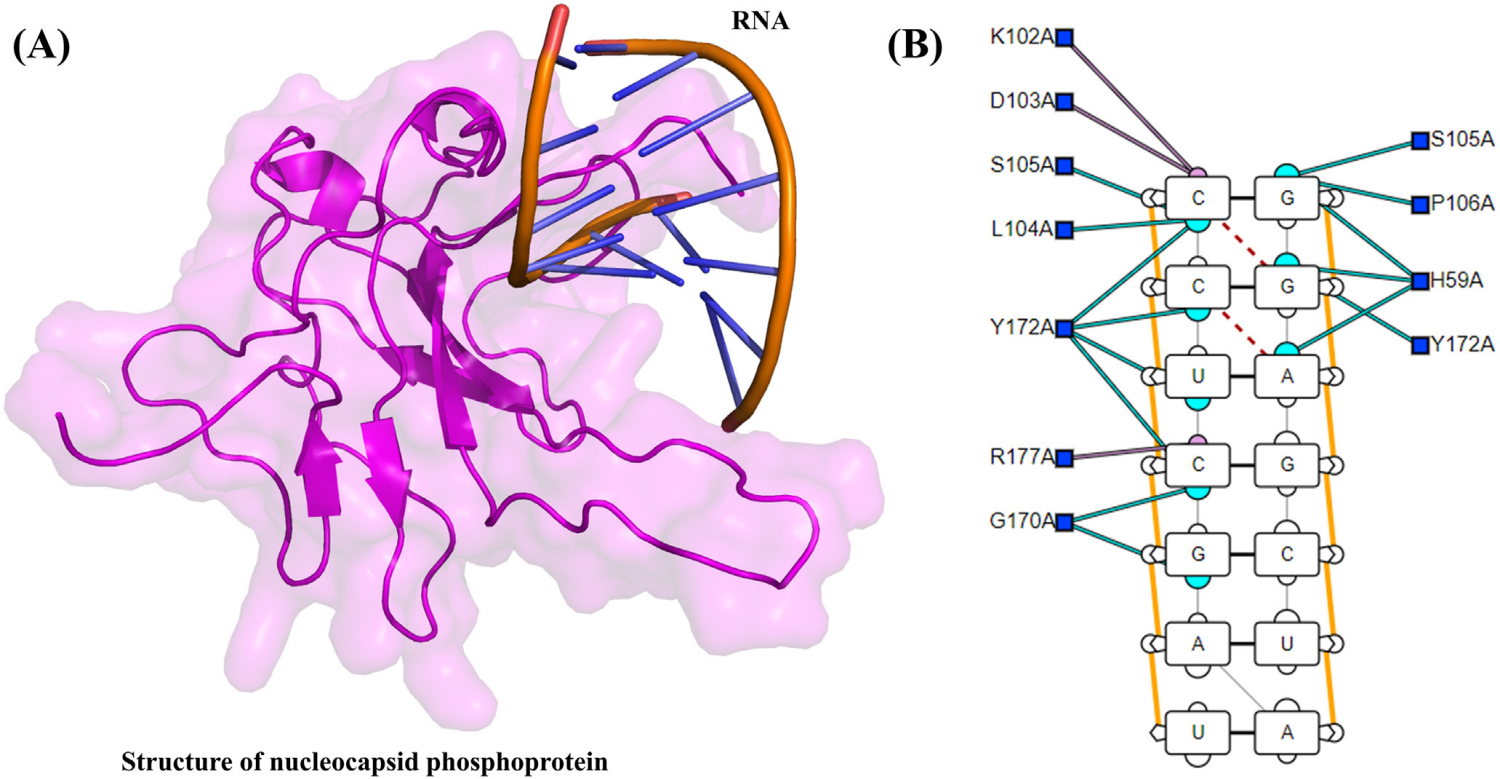
(A) Nucleocapsid phosphoprotein-RNA complex. The magenta color shows the N-terminal of Nucleocapsid phosphoprotein, while the ladder shape shows the RNA bound to the Nucleocapsid phosphoprotein of SAR-COV-2. (B) Showing the interaction of RNA with the Nucleocapsid phosphoprotein N-terminal. Thr57, His59, Ser105, Arg107, Gly170, Phe171, Tyr172 were reported to be high binding residues. (For interpretation of the references to color in this figure legend, the reader is referred to the web version of this article.)

A recently reported NMR structure of the N-terminal domain of the SARS-CoV-2 nucleocapsid phosphoprotein was retrieved from RCSB using the PDB ID: 6YI3 reported by Dinesh et al. [15]. The obtained NMR structure and the modeled RNA was submitted to HADDOCK and NPDock for molecular docking. Protein-RNA docking by HADDOCK and NPDock ranked the best conformation of nucleocapsid phosphoprotein-RNA complex (Fig. 1(A)). The total binding affinity −108.0 kcal/mol was reported for the best conformation. To understand the interaction pattern, these complexes were subjected to the DNAproDB server. This server mapped the interactions, and the results are shown in Fig. 1(B). Results from these analyses revealed that residues Thr57, His59, Lys61, Lys102, Asp103, Leu104, Ser105, Arg107, Lys169, Gly170, Phe171, Tyr172, Ala173, Gly175, Ser176, and Arg177 was detected, interacting with the RNA of SAR-CoV-2. Ribose sugar of Adenine (A), uracil (U), and Guanine (G) formed interactions with His59, Lys61, and Tyr172. The majority of the residues were interacting with the phosphate (P) group of nucleotides (Arg107, Ala173, Gly175, Ser176, Arg177, Lys169, Gly170, and Phe171). These interactions were detected with U, A, G, C, and U from 5′ end on the left side and Lys61 from the right side.

### 3.2 In-silico mutagenesis of interfacial residues

A computational mutagenesis approach was used to determine the impact of each alanine substitution. Alanine substitution defines the role of a specific residue to the stability or function of a given protein. Due to its distinguishing features like chemically inert, non-bulky, and methyl functional group attachment, alanine is considered as the best choice to calculate the impact of each residue. Herein, using multiple algorithms, the significance of each interacting residue was determined. Among the total 19 interactions, utilizing the dStability, dAffinity, and change in the binding affinity, ten substitutions were reported to increase the stability. While using the defined criteria, nine substitutions including T51A, H59A, K61A, S105A, R107A, K169A, G170A, F171A, and Y172A, was found to reduce the stability and binding affinity upon substitution. However, substitutions reported by all three tools, including MOE, DrugScorePPI, and mCSM-NA, were selected for further analysis. Using this criterion, two substitutions K61A and K169A, were excluded. The remaining seven substitutions significantly affected the binding and stability of the Protein-RNA complex. Among these four substitutions, T57A, H59A, G170A, and F171Achanged the protein-RNA in the greater fold. While the other substitutions such as S105A, R107A and Y172A were reported to affect the protein-RNA complex comparatively in the lower fold. As shown in Table 1, the seven substitutions, which reduce the stability, were selected for molecular dynamics simulation and post-simulation analysis to understand the dynamics of these substitutions.

**Table 1:** The table contains a list of interacting residues. Based on the dAffinity, dStability, and Predicted ΔΔG was used to understand the impact of each substitution when changed to alanine. The significant substitutions which reduce the binding affinity and stability of the Protein-RNA complex are given in bold.

Index	Residue Position	dAffinity	dStability	Predicted ΔΔG	Outcome
1	D103A	−0.218208126	0.7058154	5.07	Increased affinity
2	E174A	−0.627432562	1.099226294	4.5735	Increased affinity
3	F171A	0.131910418	2.094553371	−8.7225	**Reduced affinity**
4	G170A	0.799354909	0.041698953	−5.559	**Reduced affinity**
5	G175A	−0.632812623	0.687884896	6.7065	Increased affinity
6	G178A	−1.332230401	0.668020025	9.135	Increased affinity
7	G60A	−0.655284048	0.079444368	0.4935	Increased affinity
8	H59A	3.80850533	0.429009885	−8.3685	**Reduced affinity**
9	K102A	1.651298071	0.815469681	1.0365	Increased affinity
10	K169A	1.957372005	−0.315900659	−1.6425	**Reduced affinity**
11	K61A	2.456900907	−0.43984319	−2.547	**Reduced affinity**
12	L104A	3.159796329	1.738786997	5.541	Increased affinity
13	P168A	0.064328214	1.125976312	0.3195	Increased affinity
14	R107A	0.831868936	1.887304986	−1.0755	**Reduced affinity**
15	R177A	6.054382342	0.310284166	4.212	Increased affinity
16	S105A	0.957764581	0.470427697	−1.6065	**Reduced affinity**
17	S176A	0.304609561	0.649918458	1.368	Increased affinity
18	T57A	0.936589745	1.111603419	−7.2075	**Reduced affinity**
19	Y172A	6.632200002	2.154790282	−0.438	**Reduced affinity**

Molecular Dynamics Simulation.

Molecular dynamics simulation of wild and seven mutant systems was performed. Different analysis such as RMSDs for stability, RMSF for residual flexibility, Total energy, Principle component Analysis for protein motions, Free energy landscape for protein states transition, DCCM for residues correlated and anti-correlated while binding free energy for the affinity of RNA toward the protein was performed. These analyses significantly increased the understanding of RNA-protein interaction.

### 3.3 Convergence of wild and mutant systems

**Fig. 2:**
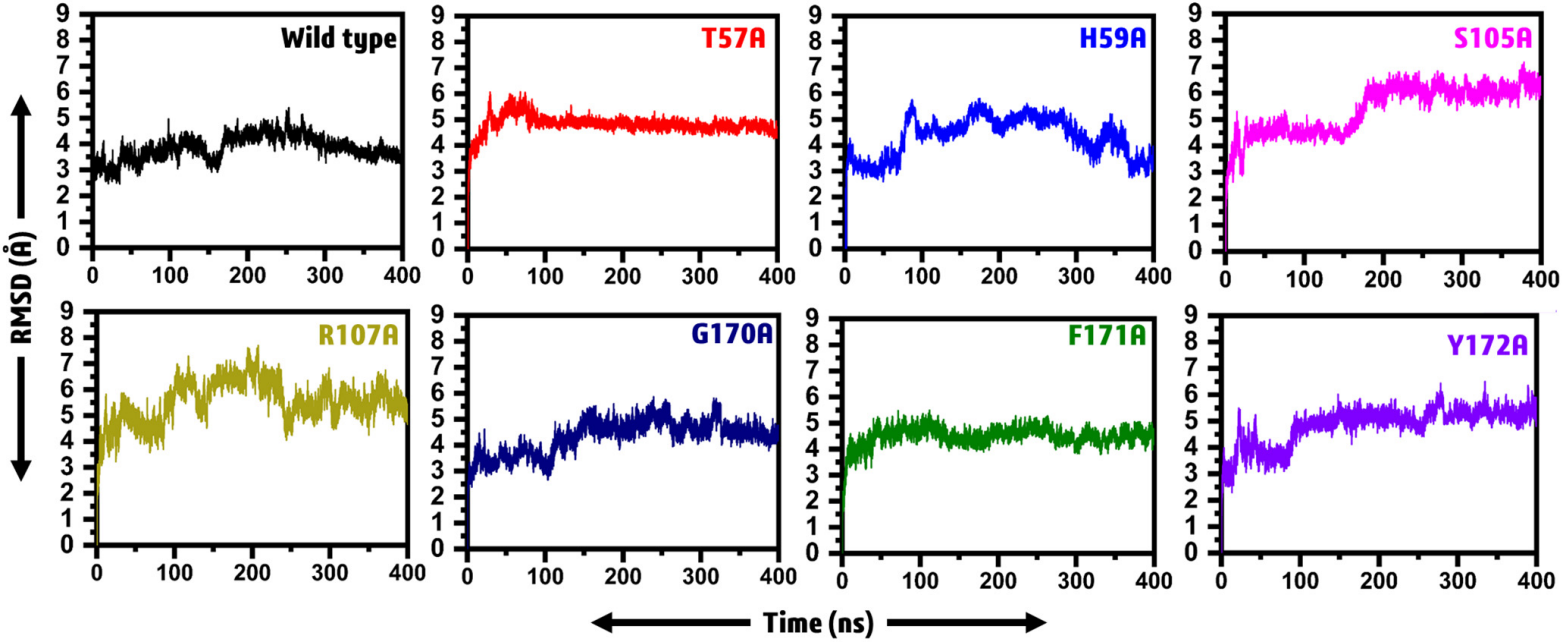
RMSDs of all the systems, including wild type and mutants. The average RMSD was reported 3.5 Å for wild type. Compare to the wild type, the average RMSD for the mutant systems were above the 4 Å.

A comparative study of MD properties on variants and the WT protein complexes was performed to check the stability of MTs during the simulation period. We repeated each simulation run three times. The trajectory was analyzed, and RMSDs were calculated after 400 ns. As given in Fig. 2, the wild type system remained stable during the course of simulation except for friction between 150 and 160 ns time period. It can be seen that the wild type system after this acceptable fluctuation has gained the stability and onward till 400 ns a straight graph is formed, which reports the stable behavior of the wild type system. In the case of the T57A mutant, the RMSD increased for the first 80 ns but remained stable for the rest of simulation time. On the other hand, H59A, which form multiple interactions with an RNA molecule, has significantly affected the overall stability of the system. From the figure, it can be explained that major convergence at different intervals occurred. Time periods between 80–100 ns, 180–200 ns, and 330–380 ns showed significant deviation during the simulation. In addition, the system S105A showed a stable graph till the 180 ns except for a substantial convergence at 180 ns time period and the RMSD increased substantially. Soon after increasing the RMSD no convergence was observed. In the case of R107A, the system showed significant deviation during the course of the simulation. Specifically, the system, R107A, showed significant convergence in the stability till the end of the simulation. Significant convergence at different intervals was observed. However, G170A, with the major stability drift between 100 and 120 ns during simulation, a continuous increase in the RMSD value was also observed. Stability fluctuation between 320 and 340 ns was also observed. In the case of F171A, the impact of alanine substitution did not favor the stability change. However, substitution Y172A significantly affected the system. The stability shifts at different intervals 50–80, 280–300 and 320–350 ns significantly affected the system's stability. Altogether, these results show that the variants T57A, H59A, S105A, R107A, G170A, F171A, and Y172 attained more deviation when compared with WT protein. MTs G170A, F171A, and Y172A were seemed unstable even at the end of the simulation period and reached a maximum RMSDs, 6 Å, 5 Å, and 4.9 Å, respectively when compared with wild type using the red line threshold. The RMSD results from the three replicates are given in Supplementary Fig. S1. It can be seen that no major differences were observed and all the simulation results are significant.

### 3.4 Root mean square fluctuation (RMSF)

**Fig. 3:**
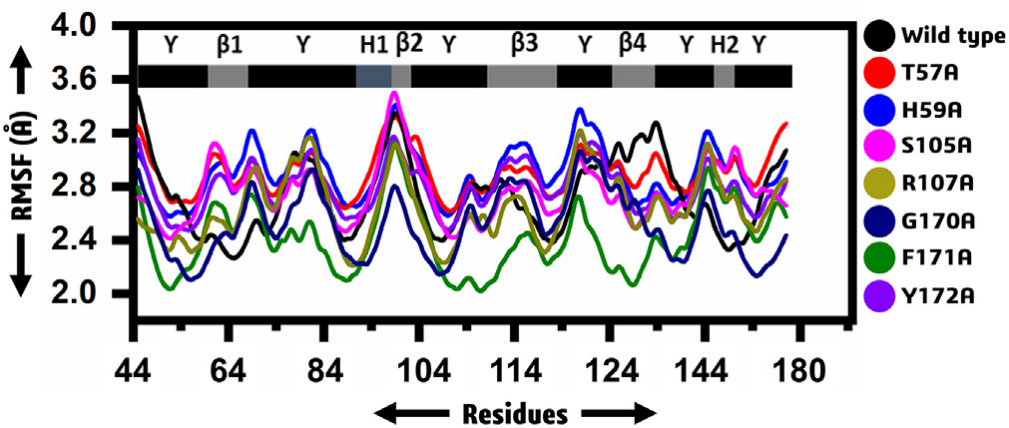
RMSF of WT and MTs. (A): WT exhibited the RMSF between 2.3 and 3.4 Å (B): T57A (C): H59A attained the highest RMSF value at the end. (D): S105A (E): R107A demonstrated RMSF between 1.1 and 2.5 Å. (F): G170F (G): F171A (H) Y17A.

The residual flexibility was calculated by mean of RMSF. It can be seen that the wild type and mutant systems exhibit more similar pattern of flexibility. The average RMSF for all the systems was observed to be 2.8 Å. As given the WT, H59, T57, S105 and R107 showed similar pattern of flexibility while the G170, F171 and Y172 possess lower flexibility than the others. The increased flexibility at different regions is due to the loops in the structure. In case of the lower flexibility shown by G170, F171 and Y172 is due to the differential dynamics upon the binding of RNA. The secondary structure given above the RMSF graph justifies the residual flexibility. Overall the residues fluctuation among MTs was detected in difference when compared with WT (Fig. 3).

**Fig. 4:**
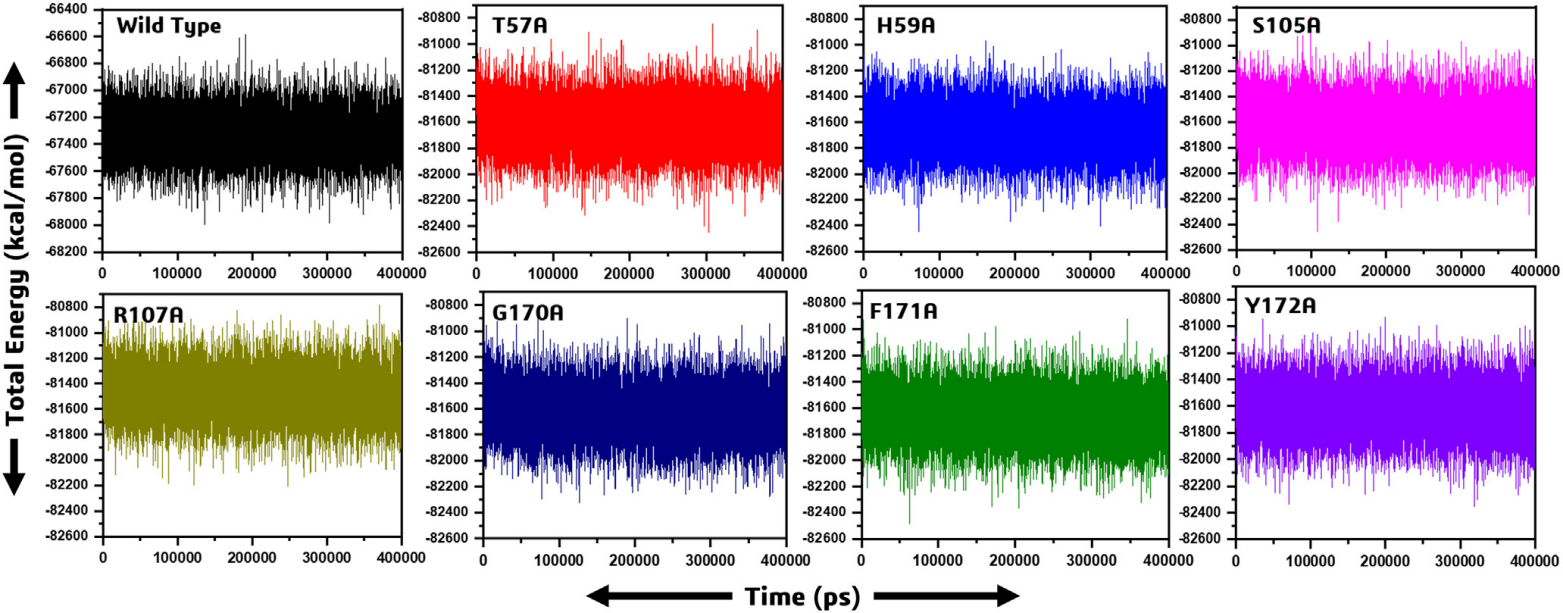
The figure shows the total energy differences between the wild and mutant systems. The x-axis is showing the time in picoseconds while the y-axis shows the total energy in kcal/mol.

The total energies of all the mutants revealed a more similar pattern ranging from −80,800 kcal/mol to −82,600 kcal/mol. On the other hand, the wild type exhibited different total energy as given in Fig. 4.

### 3.5 Clustering of proteins motion trajectories

**Fig. 5:**
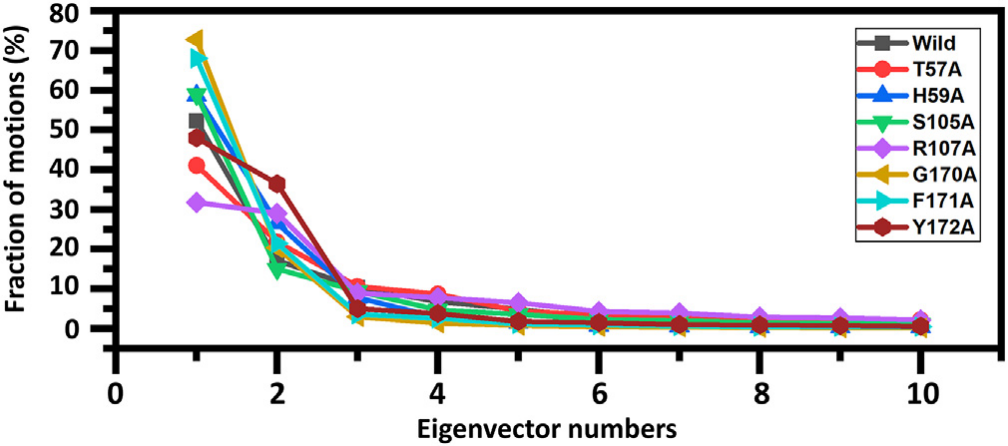
Fraction of the first 10 eigenvectors. The (%) contribution of each eigenvector obtained from covariance matrix plotted against the corresponding eigenvector indices constructed from the MD trajectory.

The impact of predicted mutations on N-NTD dynamics could be observed in Fig. 5. PCA (Principal component Analysis) was used to understand the structural changes with amplitude in each system levied by specific substitution. As given in Fig. 5, it can be seen that significant dominant motions were observed in the first three eigenvectors while the rest showed localized fluctuation. It can be seen that the first three eigenvectors contributed a total of 52% variances to the total observed motions in the wild type system. Unlikely the wild type, in mutants different behaviour of motion was observed. For each mutant 41% (T57A), 58% (H59A), 58% (S105A), 31% (R107A), 72% (G170A), 68% (F171A) while 48% (Y172A) total motion was observed. This behavior may explain the structural rearrangement due to the RNA binding.

**Fig. 6:**
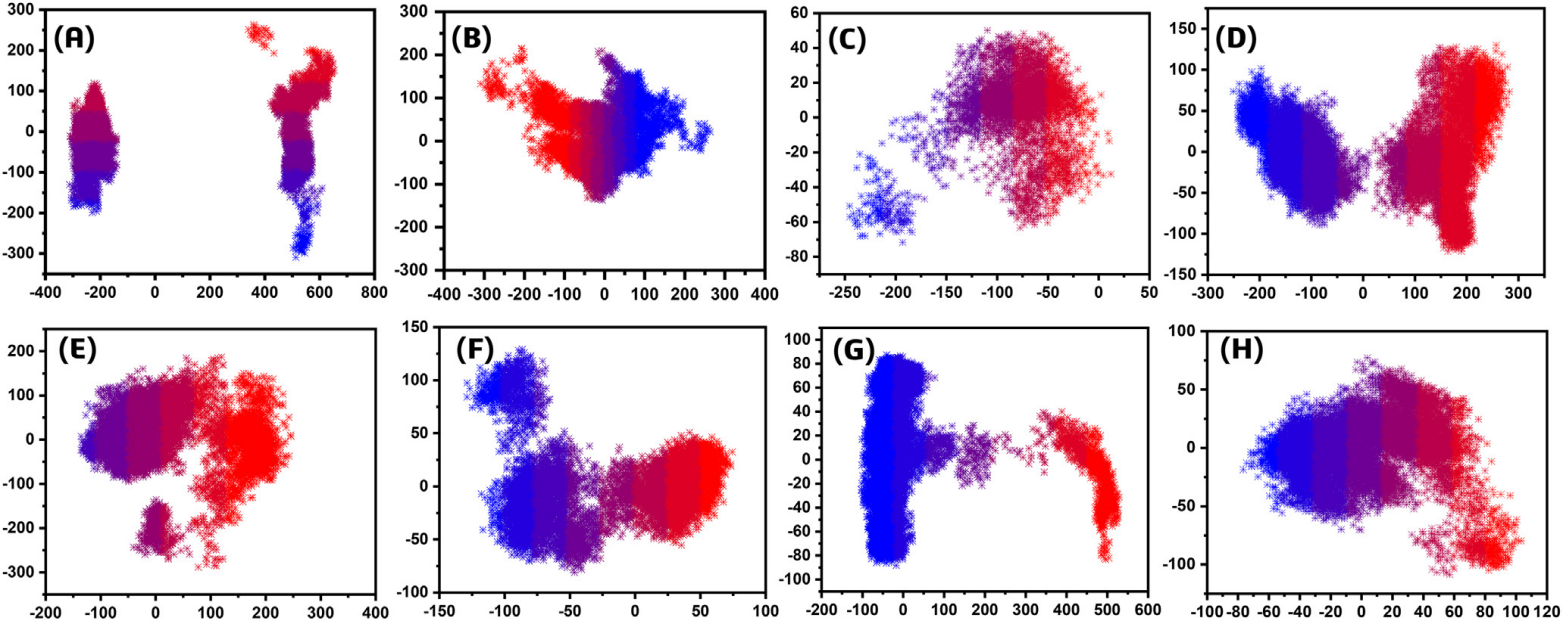
Principal component analysis (PCA) of WT and MTs N-NTD of SARS-CoV-2. (A) WT (B) T57A (C) H59A (D) S105A (E) R107A (F) G170A (G) F171A (H) Y172A. The first PC1 and second PC2 from the PCA of the backbone carbon were used.

Furthermore, to obtain conceivable attributed motions, the first two eigenvectors were plotted against each other. The depiction of the blue to red color indicates the flipping over of conformations during the simulation period. Each dot starting from blue and ends at red represent specific frame. Trajectories have been mapped into a two-dimensional subspace using PC1 and PC2 to grasp the complexes conformational transformations. It can be seen that all the complexes attained two conformational states on the subspace differently colored (blue and red) Fig. 6. These two conformational states could be easily separated from each other as the energetically unstable conformational state blue neared convergence and attaining a stable conformational state red color. Consequently, different periodic jumps are required for the transition of different conformations in mutants.

### 3.6 Transition pathway from metastable to native states

**Fig. 7:**
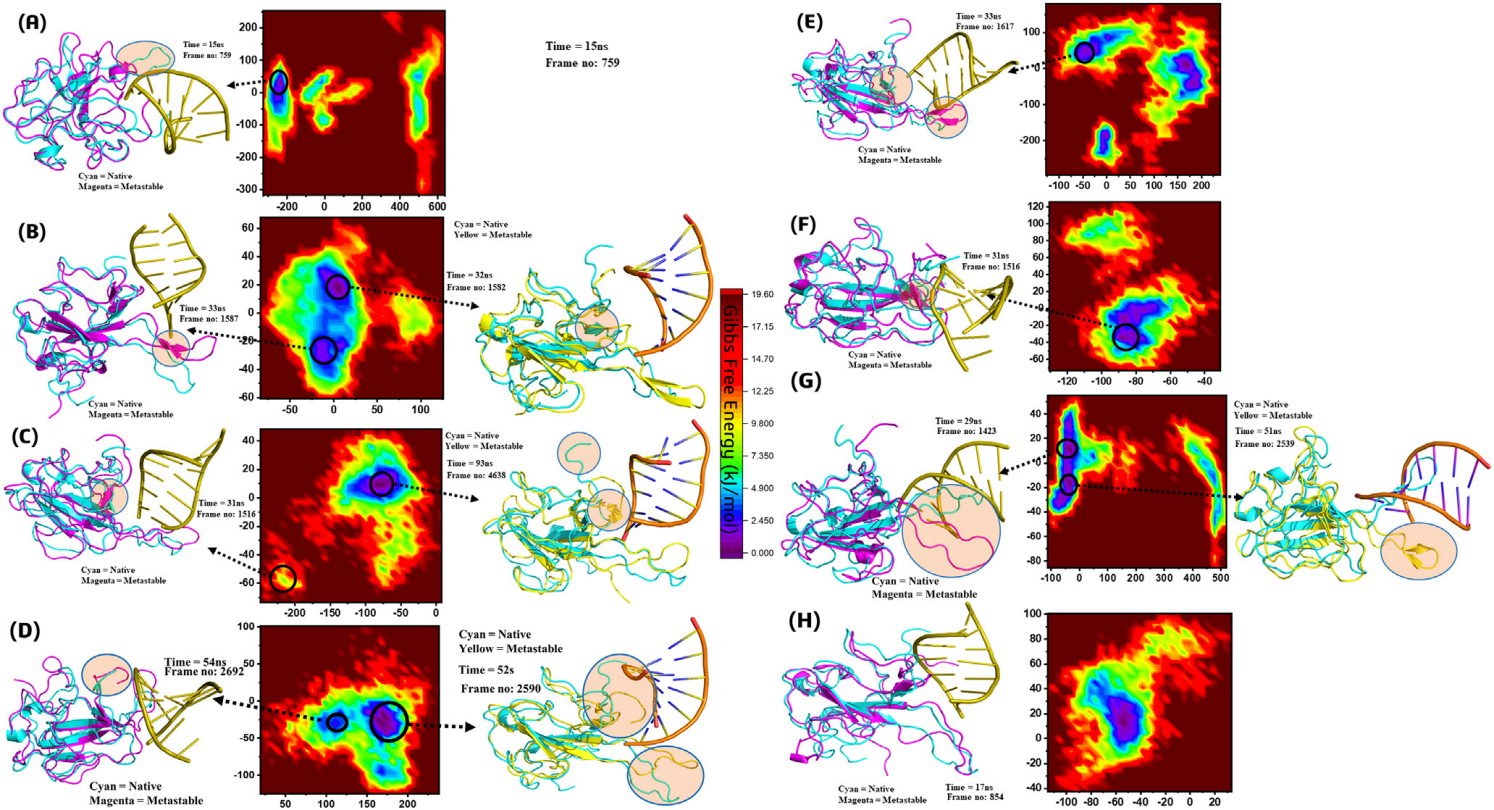
Free energy landscape (FEL) of Wild type and Mutants. High and low energy state has been represented by a different color in the plot. The contour scale is given and the dark colour represent each minimal energy structural ensemble. Red shows a high energy state. Yellow shows an intermediate energy state (A, B, C, D, E, F, G, H) represent the conformational transition states in MTs, T57A, H59A, S105A, R107A, G170A, F171A, and Y172A. (For interpretation of the references to color in this figure legend, the reader is referred to the web version of this article.)

The free energy landscape (FEL) depicts the transition states. To understand the transition mechanism of MTs and WT complexes from metastable to native states, the first two eigenvectors were considered for computing and plotting the FEL of trajectory time. For better understanding the structural evolution, low energy states have been mined. WT demonstrated a significant difference in FEL when compared with MTs, as shown by colors in the plot **(**Fig. 7**)**. The color red is more prevalent in MTs (T57A, H59A, S105A, R107A, G170A, F171A, and Y172A), seems unstable compared to WT. The highest transition states have been observed in H59A, S105A, R107A, and F171A, showing the impact of these residues’ mutation on RNA bindings. WT exhibited two states and separated by an energy barrier. It can also be seen that the WT remained in one energy state for most of the time. G170A and Y172A also attained a more intermediate state (yellow). However, the difference is evident between WT and MTs, depicting the impact of these mutations on FEL. The result specifies the more conformational transition in the MTs compared to WT. Multiple metastable states have been observed in MTs during their structural evolution. These have been separated by low and high-energy barriers**.** The changes in different structural ensemble at different time nanosecond are given in cartoon structures while the critical regions are shaded. The x and y coordinates, their respective frame number and time (ns) is given in Supplementary Table S5.

### 3.7 Dynamical cross-correlated map analysis for wild & mutant systems

**Fig. 8:**
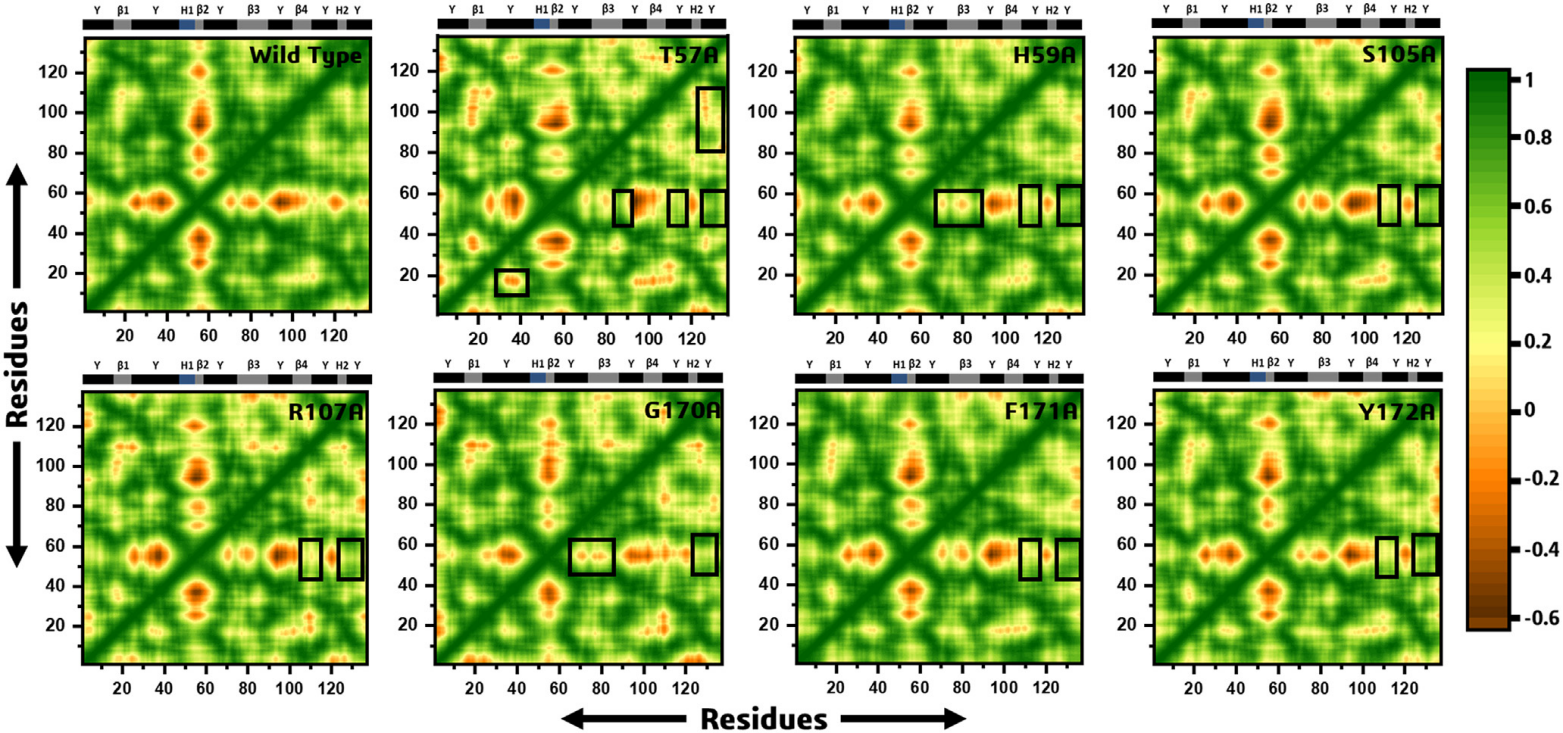
Dynamic cross-correlation (DCCM) plot of WT and MTs. The colors show the positive and negative correlated motions of residues in WT and MTs complexes. The color code at the right represents the quantity of positive and negative correlation. A more reddish represents negatively correlated motion among residues. The color inclines characterize a gradual decrease in the correlation motion.

To investigate the functional displacements of the interaction protein atoms as a function of time, we constructed and analyzed a dynamics cross-correlation matrix (DCCM). During the simulation time (400 ns) wild type showed a more positive correlated motion with a negative strand correlation of loop (ϒ2). All mutants demonstrated variation in correlated motions where the maximum of the residues exhibited positive correlations than wild type complex. All the correlation plots are given in Fig. 8.

It can be seen that overall the motions are dominated by the correlated motions. In case of the T57A when compared to the wild type the loop (ϒ1) showed a negative correlation while the β3, ϒ5 and ϒ6 showed a positive correlation. On the other hand, H59A showed a positive correlation at ϒ5 region. Here a weak negative correlation at β3 site was observed. S105A possess strong negative correlation except the regions ϒ5 and ϒ6, which showed a positive correlation when compared to the wild type. R107A showed positive correlation at regions β4 and ϒ2 while the rest a similar pattern was observed. Furthermore, G170A showed a weak negative correlation at regions where the wild type showed strong negative correlation. The region ϒ6 was reported to possess positive correlation. In case of F171A and Y172A a more similar pattern of correlation was observed where the region ϒ6 showed strong positive correlation while the other regions showed strong negative correlation. Thus, the substitutions affect the internal dynamics of the interacting proteins and ultimately the biology of binding with the RNA. These results signify that the substitution has brought conformational and dynamical variability and, therefore discloses the structure–function relationship specifically the affinity for binding the RNA molecule.

### 3.8 Binding free energy calculations

Free energy computation and analysis were performed to compare the interaction changes in wild type and mutant systems quantitatively. To compute the total free energy, we used 20,000 snapshots from the last 400 ns of the MD simulation trajectory. Both MMGBSA and MMPBSA for each run (three replicates) were calculated. Each contributing term such as van der Waals (vdW), electrostatic, polar solvation, and solvent accessible surface area (SASA) energies were calculated and are given in Table 2 (MMGBSA) and Table 3 **(**MMPBSA). The MMGBSA and MMPBSA results for replicate 2 and replicate 3 are given in Supplementary Table S1–S4.

**Table 2:** MM-GBSA of wild type and mutant systems.

Complex Name	MMGBSA (kJ/mol)				
	Δ_vdW_	Δ_elec_	Δ_ps_	Δ_SASA_	Δ_G Total_
Wild Type	−1186.03 ± 19.85	−9421.01 ± 124.93	−3486.63 ± 117.85	73.67 ± 1.90	−6426.53 ± 42.24
T57A	−1190.13 ± 19.07	−9232.48 ± 203.81	−3367.82 ± 197.96	72.21 ± 2.25	−6007.59 ± 49.09
H59A	−1184.39 ± 25.84	−9142.96 ± 141.49	−3450.93 ± 126.22	72.83 ± 3.16	−5924.87 ± 44.57
S105A	−1193.50 ± 19.23	−9163.89 ± 133.27	−3391.34 ± 114.43	72.88 ± 1.63	−5969.11 ± 39.57
R107A	−1175.41 ± 17.86	−8995.88 ± 191.93	−3600.40 ± 191.60	74.39 ± 1.88	−5904.09 ± 42.92
G170A	−1181.64 ± 19.94	−9208.50 ± 127.29	−3416.87 ± 10.22	74.18 ± 2.31	−5812.52 ± 53.52
F171A	−1156.73 ± 21.58	−8968.67 ± 125.18	−3719.97 ± 124.34	77.14 ± 2.31	−5311.79 ± 46.67
Y172A	−1189.97 ± 17.84	−9068.05 ± 109.49	−3503.93 ± 96.30	73.08 ± 1.97	−5985.77 ± 38.43

**Table 3:** MMPBSA of wild type and mutant systems.

Complex Name	MMPBSA (kJ/mol)			
	Δ_vdW_	Δ_elec_	Δ_ps_	Δ_G Total_
Wild	−51.99 ± 11.23	−1601.34 ± 102.01	1668.063 ± 108.13	−41.54 ± 12.30
T57A	−31.28 ± 12.66	−1317.27 ± 94.28	1227.43 ± 169.48	−35.59 ± 38.21
H59A	−47.58 ± 16.14	−1405.26 ± 201.36	1180.23 ± 98.691	−33.22 ± 15.38
S105A	−53.56 ± 11.63	−1215.61 ± 121.15	1512.22 ± 83.58	−32.41 ± 11.20
R107A	−49.53 ± 10.03	−1447.19 ± 132.12	1407.13 ± 101.02	−31.88 ± 9.36
G170A	−32.41 ± 12.32	−1367.22 ± 142.24	1347.06 ± 131.78	−32.85 ± 11.69
F171A	−51.02 ± 12.57	−1321.45 ± 121.47	1212.21 ± 86.63	−27.56 ± 14.28
Y172A	−57.28 ± 12.21	−1127.54 ± 104.15	1107.18 ± 29.65	−33.93 ± 10.06

**Elec** = electrostatic energy; **G-Total** = total binding free energy; **Ps** = polar solvation energy; **SASA** = solvent‐accessible surface area energy; Δ**vdW** = van der Waals energy; **MMGBSA** = Molecular Mechanics/Generalized Born Surface Area.

The MM-GBSA results (Table 2) also reveals variation in energies among WT and MTs. In majority, this effect is high in terms of total and electrostatic energies. WT exhibited the ΔvdW (−1186.03 ± 19.85 kj/mol) Δelec (9421.01 ± 124.93 kj/mol) Δps (−3486.63 ± 117.85 kj/mol) ΔSASA (73.67 ± 1.90), and ΔG Total energies (−6426.5 ± 42.2) which have been found in variation with MTs except R107A, G170A, and F171A The vdW energy was in less variation between WT −1186.03 ± 19.85 kJ/mol) and MTs, T57A, H59A, S105A, R107A, G170A, F171A, and Y172A (Table 2). While differences in electrostatic energies between WT and MTs complexes is significantly high., suggesting that these locations might be essential for binding RNA through electrostatic interactions with N-NTD of SARS-CoV-2. SASA energy of WT has not been observed in significant variations except R107A, G170A, and F171A (74.39 ± 1.88kj/mol, 74.18 ± 2.31kj/mol, and 77.14 ± 2.31kj/mol) where the SASA energy is higher than that WT, suggesting the impact of alanine mutations on binding with virus RNA and N-NTD SASA energy.

The MM/PBSA results shows that WT-RNA complex exhibited the highest binding energy 41.54 ± 12.30), as compared to the MTs complexes (Table 3). The highest impact on the total binding energy was found in F171A and R107A, −27.56 ± 14.28 and −31.88 ± 9.36 (kJ/mol). The vdW of WT and MTs has been found in variation where the WT attained the lowest energy state (−51.99 ± 11.23 kj/mol) when compared with MTs. The lowest electrostatic energy has been attained by WT (−1601.34 ± 102.01 kj/mol), however, MTs H59A, R107 also attained a good **Δ**elec energy as shown in Table 1. Potential energy has been found in significant difference except, signifying the effect of mutations on structure and interaction with RNA.

### 3.9 Per-residue energy decomposition analysis

**Fig. 9:**
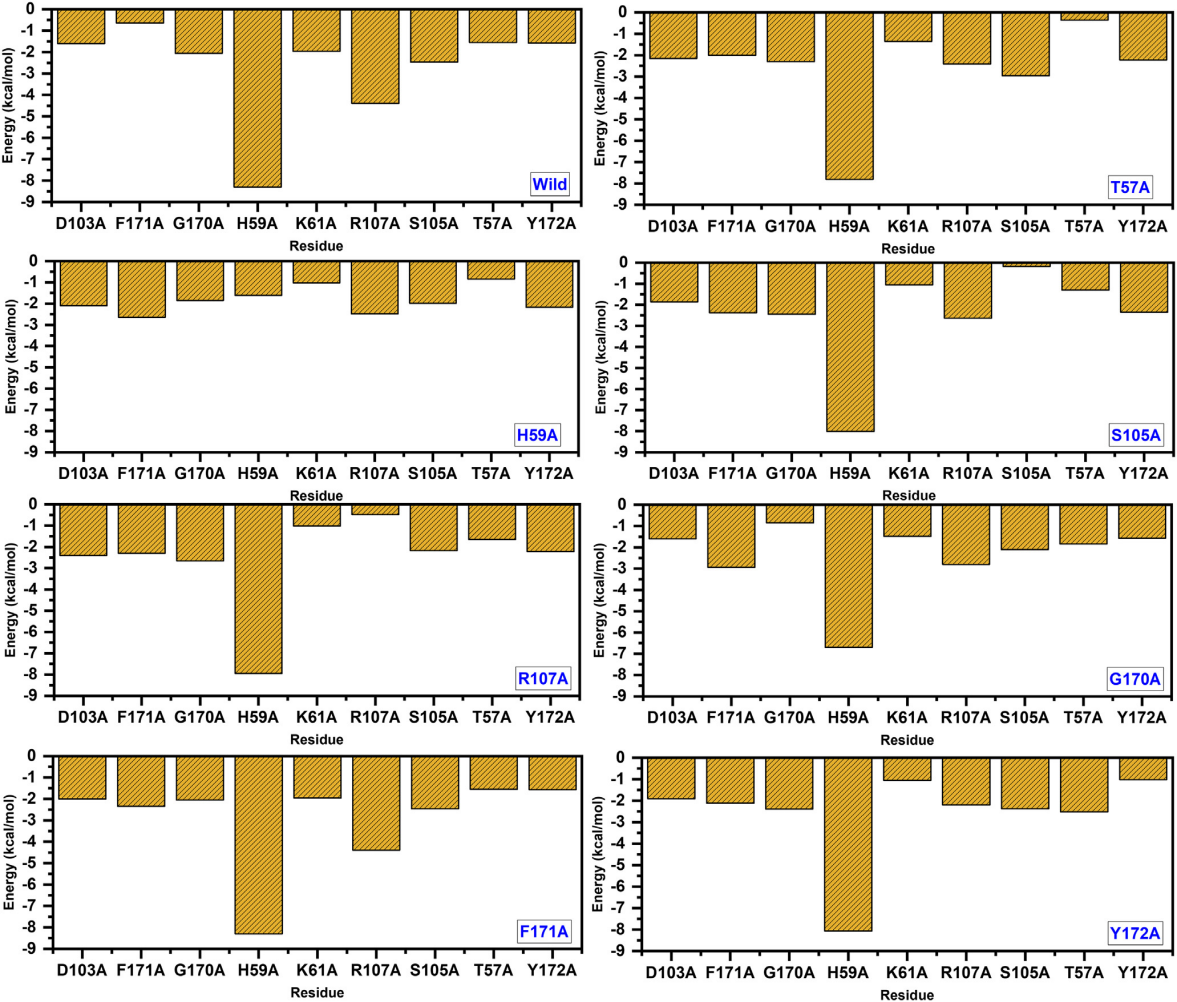
Per-residue energy decomposition analysis of the essential residues contributes to the total binding energy.

Furthermore, to understand the impact of each residue on the binding of RNA we calculated the energy contribution from each residue to the total energy. Our analysis suggests that among the seven residues T57, H59, S105 and R107 contributes more to the total energy. As given in Fig. 9, it can be seen that H59 contributes the most followed by R107, S105 and T57. Hence these results confirm that while designing small molecule inhibitors these residues should be the primary targets. We speculate the blocking these residues could help to block the SARs-CoV-2 pathogenicity.

## 4 Discussion

The nucleocapsid phosphoprotein (N) is playing a role in linking the viral + RNA to the membrane. There are two domains, N-terminal RNA binding domain (N-NTD) that binds the RNA. In contrast, the C-terminal domain (CTD), after interaction with the M protein, is involved in anchoring the ribonucleoprotein to the viral membrane [42]. Although the previous study [15] unveil that RNA binding to N-NTD and its interaction with RNA, however, the mechanism and the impact of mutation has not been yet investigated. Here in the current investigation, we performed comprehensive MD simulation to unveil the binding mechanism, types of interactions, and the impact of mutations on N proteins’ dynamic behavior. Residues T57, H59, S105A, R107A, G170, F171, Y172 have been found, playing a significant role in interaction with RNA. A more recent study also reported that amino acid residues A50, T57, H59, R92, I94, S105, R107, R149, Y172 are essential in the establishment of interactions with SARS-CoV-2 RNA (Dinesh et al. 2020). The molecular mechanisms to recognize RNA binding N protein and the establishment of interactions will increase our understating to design future inhibitors. Our protein model docking, and simulation analysis exposed that N-NTD recognizes and establishing contacts in a shape-specific manner by with RNA. The same results have been described earlier, where stem-loop mRNA is recognized by adenosine deaminase RNA specific 2 (ADAR2) [43]. Previous studies demonstrated that residues S105 and R107 are conserved among all SARS-CoV N-NTD (SARS-CoV-2, SARS-CoV, MERS-CoV, and HCoV-OC43) [4]. Mutating S105 and R107 results in the incapability of p4a of blocking IFN production in cells infected within MERS-CoV (Siu et al. 2014). Remarkably, we detected that S105 and R107 residues retained contacts with RNA when subjected to 400 ns MD simulations. Mutating these residues in alanine scanning results in a significant impact on N-NTD structure dynamic behavior and interactions with RNA binding. These findings further support the results of previous reports and propose to design inhibitors against these residues playing a vital role in N-NTD-RNA interaction in SARS-CoV-2 that may be helpful for better management of COVID-19 infections. To validate the role of residues involved in an interaction with RNA, Rigorous in silico alanine scanning and MD simulations was performed for a period of 400 ns to pinpoint the role these residues and their impact on dynamics and free energy calculations where residues T57A, H59A, S105A, R107A, G170A, F171A, and Y172A were found, influencing the binding affinity between SARS-CoV-2N-NTD and RNA binding. Inhibitors may be designed to block the RNA interactions site. Alanine scanning is a reliable approach in predicting residues at protein interfaces that might be involved in binding with ligands with potential for modulation [44]. Binding of drugs or other biomolecules at protein interfaces is mostly controlled by some specific residues contributing disproportionately to the Gibbs free energy of binding, ΔG, and dynamics of proteins, which are good targets for drug designing and discovery. The trajectory investigation through RMSD, RMSF, and essential dynamics showed that variants created, displayed variations in the 3D structure of SARS-CoV-2N-NTD that might affect the affinity towards RNA. These variants exhibited marked significant impact in RMSD, RMSF, DCCM, and PCA. All the alanine variants established a discrete pattern of structural dynamics and very interesting because point mutations have been created in the same crystal structure (WT) and compared during the whole investigation. In simulated or natural conditions, the substitution with alanine is sufficient to cause variations in protein structural dynamics, affecting binding capability. The binding free energy demonstrated that N-NTD exhibited a decreased affinity toward RNA in MTs T57, H59, S105A, R107A, G170, F171, and Y172. Since these methods are widely used by different studies to understand the impact of mutations [45], [46].

In conclusion, residues T57, H59, S105, R107, G170, F171, and Y172 are playing a significant role in binding with RNA of SARS-CoV-2. Alanine scanning further supported the role of these residues when subjected to comprehensive MD simulation. The overall structural dynamics, including RMSD, RMSF, DCCM, and PCA, have been found, influenced by alanine MTs. Binding free energy further supported that these residues might have a role in binding with RNA. Drug development and screening against these residues may be useful for better management of SARS-CoV-2 infections. The fluctuations and changes observed in the longer and repeated simulation could provide better understanding. The observed variations in different replicas are significantly correlated and could aid to design small molecule inhibitors which could target the N-terminal domain of SARs-CoV-2N-NTD protein and may halt the RNA recognition to aid the treatment process.

## Authors contribution

AK, MTK, SS and MJ, conceptualized the study and did the analysis. AA, SSA, MK wrote the manuscript. AA, SSA and MK also contributed to the methodology. AK revised the manuscript and did all the additional analysis. AK contribution is major. DQW is an academic supervisor. He supervised the study.

## Declaration of Competing Interest

The authors declare that they have no known competing financial interests or personal relationships that could have appeared to influence the work reported in this paper.
