# Supplementary material for: Structural insights into the mechanism of RNA recognition by the N-terminal RNA-binding domain of the SARS-CoV-2 nucleocapsid phosphoprotein
Source: Comput Struct Biotechnol J. 2020 Aug 12;18:2174–84. doi: 10.1016/j.csbj.2020.08.006 (PMC7419326; doi:10.1016/j.csbj.2020.08.006)
Supplement: Supplementary data 1 [file mmc1.docx]

**Structural Insights into the mechanism of RNA recognition by the N-terminal RNA-binding domain of the SARS-CoV-2 nucleocapsid phosphoprotein**

Abbas Khan^1#^, Muhammad Tahir Khan^1#^, Shoaib Saleem^2#^, Muhammad Junaid^1^, Arif Ali^1^, Syed Shujait Ali^3^, Mazhar Khan^4^, Dong-Qing Wei^1,5,6*^

^1^State Key Lab of Microbial Metabolism, Department of Bioinformatics and Biological Statistics, School of Life Sciences and Biotechnology, Shanghai Jiao Tong University, Shanghai, 200240, China.

^2^National Center for Bioinformatics, Quaid-i-Azam University, 45320 Islamabad Pakistan.

^3^Center for Biotechnology and Microbiology, University of Swat, Swat, Khyber Pakhtunkhwa, Pakistan.

^4^The CAS Key Laboratory of Innate Immunity and Chronic Diseases, Hefei National Laboratory for Physical Sciences at Microscale, School of Life Sciences, CAS Center for Excellence in Molecular Cell Science, University of Science and Technology of China (USTC), Collaborative Innovation Center of Genetics and Development, Hefei, 230027, Anhui, China.

^5^State Key Laboratory of Microbial Metabolism, Shanghai-Islamabad-Belgrade Joint Innovation Center on Antibacterial Resistances, Joint Laboratory of International Cooperation in Metabolic and Developmental Sciences, Ministry of Education and School of Life Sciences and Biotechnology, Shanghai Jiao Tong University, Shanghai 200030, P.R. China

^6^Peng Cheng Laboratory, Vanke Cloud City Phase I Building 8, Xili Street, Nashan District, Shenzhen, Guangdong, 518055, P.R China.

**^#^Contributed Equally**

**Corresponding author***

**Dong-Qing Wei**

[**dqwei@sjtu.edu.cn**](mailto:dqwei@sjtu.edu.cn)


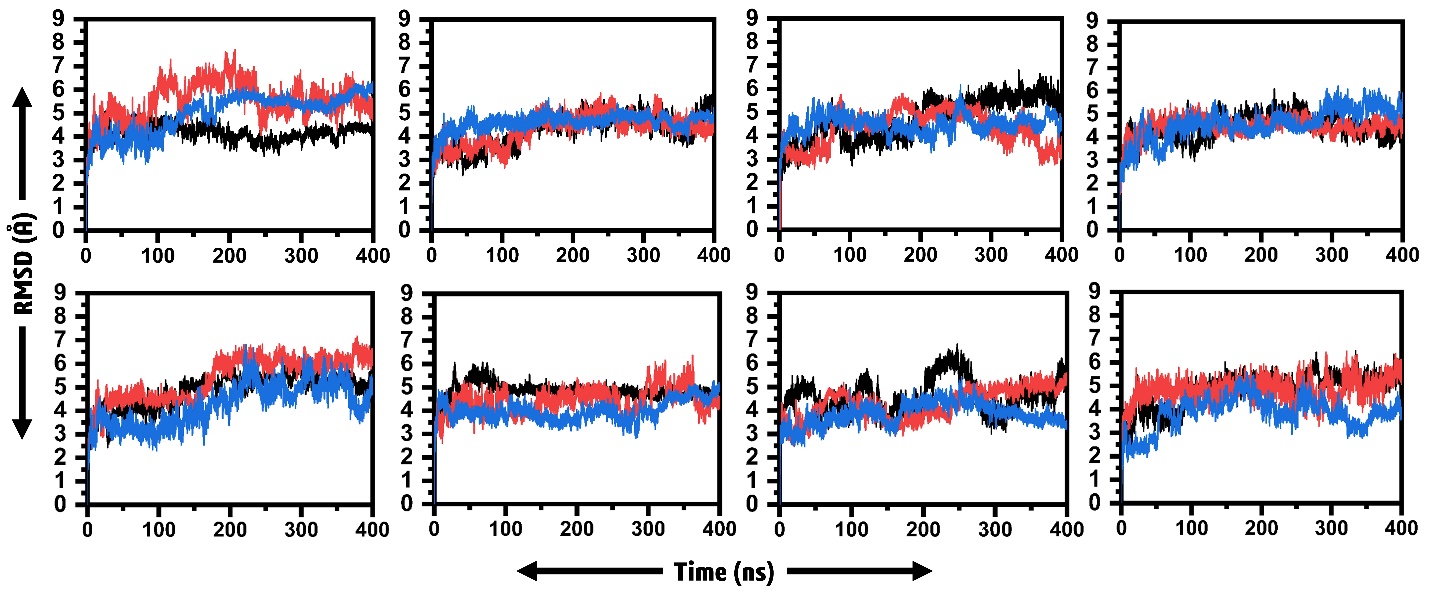


**Supplementary Figure S1:** RMSDs of all the systems repeated three times.

**Supplementary Table S1:** MMGBSA results of replicate 2.

| Complex Name | MMGBSA (kJ/mol) | | | | |
| --- | --- | --- | --- | --- | --- |
|  | **Δ_vdW_** | **Δ_elec_** | **Δ_ps_** | **Δ_SASA_** | **Δ_G Total_** |
| Wild | -1188.61±19.53 | -9351.33±123.90 | -3222.89±114.10 | 78.38±1.79 | -6317.18±40.76 |
| T57A | -1180.97±21.19 | -9192.61±151.76 | -3458.66±132.50 | 73.60±2.16 | -6016.88±45.32 |
| H59A | -1162.58±19.45 | -9094.44±147.58 | -3490.63±132.73 | 75.64±2.11 | -5988.91±44.51 |
| S105A | -1192.90±22.79 | -9090.61±159.36 | -3597.59±165.26 | 72.75±2.76 | -5827.88±44.63 |
| R107A | -1202.90±16.79 | -9165.40±127.63 | -3453.58±115.18 | 72.63±1.81 | -5908.30±41.17 |
| G170A | -1183.15±26.00 | -9066.33±232.31 | -3523.87±257.38 | 75.72±2.75 | -5706.46±41.99 |
| F171A | -1182.90±12.76 | -9008.67±149.52 | -3512.25±167.66 | 71.98±2.49 | -5331.84±42.31 |
| Y172A | -1174.32±20.59 | -9071.99±133.12 | -3506.80±112.27 | 74.07±2.36 | -5976.27±45.03 |

**Supplementary Table S2:** MMPBSA results of replicate 2.

| Complex Name | MMPBSA (kJ/mol) | | | |
| --- | --- | --- | --- | --- |
|  | **Δ_vdW_** | **Δ_elec_** | **Δ_ps_** | **Δ_G Total_** |
| Wild | -32.25±5.64 | -1283.40±101.79 | 1282.23±99.088 | -38.56±8.39 |
| T57A | -41.04±11.19 | -1380.40±201.62 | 1382.78±201.62 | -34.06±13.06 |
| H59A | -33.95±15.24 | -1228.34±87.36 | 1234.40±84.60 | -33.05±11.24 |
| S105A | -56.02±7.19 | -1430.67±114.40 | 1471.87±113.78 | -31.32±10.35 |
| R107A | -33.62±9.55 | -1040.91±121.83 | 1045.92±124.50 | -32.93±8.78 |
| G170A | -38.44±10.80 | -1306.08±117.75 | 1314.61±121.92 | -34.76±9.88 |
| F171A | -29.36±12.06 | -1173.97±179.56 | 1180.67±181.61 | -26.16±9.39 |
| Y172A | -65.98±6.40 | -1267.93±94.74 | 1296.18±91.20 | -34.74±9.66 |

**Supplementary Table S3:** MMGBSA results of replicate 3.

| Complex Name | MMGBSA (kJ/mol) | | | | |
| --- | --- | --- | --- | --- | --- |
|  | **Δ_vdW_** | **Δ_elec_** | **Δ_ps_** | **Δ_SASA_** | **Δ_G Total_** |
| Wild | -1157.23±16.82 | -9483.01±121.33 | -3442.45±116.13 | 73.18±1.14 | -6379.18±40.76 |
| T57A | -1183.25±19.11 | -9202.61±153.27 | -3388.59±130.05 | 73.26±2.06 | -6001.24±47.13 |
| H59A | -1171.33±18.12 | -9121.18±144.10 | -3514.33±128.43 | 74.44±2.01 | -5994.11±44.25 |
| S105A | -1189.77±20.10 | -9101.47±143.42 | -3512.41±159.88 | 73.01±2.40 | -5861.36±41.20 |
| R107A | -1192.26±18.86 | -9112.40±123.25 | -3453.58±163.15 | 72.02±1.29 | -5912.27±53.22 |
| G170A | -1180.09±19.80 | -9111.23±198.42 | -3420.41±214.00 | 72.18±2.36 | -5763.19±33.25 |
| F171A | -1163.41±15.48 | -8893.65±136.36 | -3493.44±153.01 | 76.93±2.01 | -5299.35±39.07 |
| Y172A | -1180.21±17.88 | -9066.65±110.38 | -3498.12±100.11 | 74.22±2.14 | -5980.33±19.83 |

**Supplementary Table S4:** MMPBSA results of replicate 3.

| Complex Name | MMPBSA (kJ/mol) | | | |
| --- | --- | --- | --- | --- |
|  | **Δ_vdW_** | **Δ_elec_** | **Δ_ps_** | **Δ_G Total_** |
| Wild | -53.54±9.55 | -1491.57±116.97 | 1515.185±115.66 | -40.04±11.65 |
| T57A | -34.07±16.86 | -1177.15±148.35 | 1181.01±146.40 | -34.24±33.46 |
| H59A | -49.70±15.24 | -1208.31±125.34 | 1227.25±129.83 | -33.76±11.24 |
| S105A | -50.77±8.85 | -1120.34±108.42 | 1145.09±115.92 | -31.79±15.68 |
| R107A | -52.41±6.23 | -1321.33±245.56 | 1354.56±131.23 | -30.46±13.21 |
| G170A | -29.26±20.71 | -1340.12±289.00 | 1341.56±297.73 | -31.54±13.74 |
| F171A | -56.81±7.25 | -1404.28±95.20 | 1409.58±95.18 | -28.93±11.06 |
| Y172A | -53.63±7.34 | -1351.51±83.46 | 1367.38±84.05 | -34.06±7.53 |

**Supplementary Table S5:** Free energy landscape results of all the systems. The table represent the X, Y coordinates, frame number and time in ns.

| **Complex Name** | **X Coordinates** | **Y Coordinates** | **Frame No** | **Time (ns)** |
| --- | --- | --- | --- | --- |
| **Wild Type** | -232.73 | 13.95 | 759 | 17ns |
| **T57A** | 35.13 | -189.48 | 1582 | 32ns |
|  | 33.69 | -203.39 | 1587 | 33ns |
| **H59A** | -76.28 | 10.30 | 4638 | 93ns |
|  | -81.10 | 9.10 | 1516 | 31ns |
| **S105A** | 170.18 | -18.41 | 2692 | 54ns |
|  | 172.92 | -21.35 | 2590 | 52ns |
| **R107A** | 169.05 | 12.23 | 1617 | 33ns |
| **G170A** | -88.31 | -34.32 | 3488 | 70ns |
| **F171A** | -34.24 | 11.07 | 1423 | 29ns |
|  | -36.30 | -16.44 | 2539 | 51ns |
| **Y172A** | -54.24 | 13.95 | 854 | 17ns |
